# Smart Textiles for Improved Quality of Life and Cognitive Assessment

**DOI:** 10.3390/s21238008

**Published:** 2021-11-30

**Authors:** Giles Oatley, Tanveer Choudhury, Paul Buckman

**Affiliations:** School of Engineering, IT and Physical Sciences, Federation University, Berwick, VIC 3805, Australia; t.choudhury@federation.edu.au (T.C.); p.buckman@federation.edu.au (P.B.)

**Keywords:** smart textiles, quality of life, gamification, cognitive appraisal, sensorized garments, mild cognitive impairment, assistive technologies

## Abstract

Smart textiles can be used as innovative solutions to amuse, meaningfully engage, comfort, entertain, stimulate, and to overall improve the quality of life for people living in care homes with dementia or its precursor mild cognitive impairment (MCI). This concept paper presents a smart textile prototype to both entertain and monitor/assess the behavior of the relevant clients. The prototype includes physical computing components for music playing and simple interaction, but additionally games and data logging systems, to determine baselines of activity and interaction. Using microelectronics, light-emitting diodes (LEDs) and capacitive touch sensors woven into a fabric, the study demonstrates the kinds of augmentations possible over the normal manipulation of the traditional non-smart activity apron by incorporating light and sound effects as feedback when patients interact with different regions of the textile. A data logging system will record the patient’s behavioral patterns. This would include the location, frequency, and time of the patient’s activities within the different textile areas. The textile will be placed across the laps of the resident, which they then play with, permitting the development of a behavioral profile through the gamification of cognitive tests. This concept paper outlines the development of a prototype sensor system and highlights the challenges related to its use in a care home setting. The research implements a wide range of functionality through a novel architecture involving loosely coupling and concentrating artifacts on the top layer and technology on the bottom layer. Components in a loosely coupled system can be replaced with alternative implementations that provide the same services, and so this gives the solution the best flexibility. The literature shows that existing architectures that are strongly coupled result in difficulties modeling different individuals without incurring significant costs.

## 1. Introduction

### 1.1. Dementia and Mild Cognitive Impairment

Dementia is the term used to describe the symptoms of a large group of illnesses that cause a progressive decline in a person’s mental functioning. It is a broad term that describes symptoms such as the loss of memory, intellect, rationality, social skills, and normal emotional reactions. Mild cognitive impairment (MCI) is considered an intermediate state between normal age-related cognitive level and dementia [1,2], and between 19 and 50% of patients with MCI progress are diagnosed with a form of dementia within 3 years. Currently, 10% percent of the population is over 60, this age group is more likely to suffer physical and cognitive impairments. Worldwide, by 2030, it is estimated that 74.7 million people (100 million by 2050) will progress to the most severe form of dementia, Alzheimer’s disease [3,4,5,6]. A systematic approach is required to assist all parties, the patient, carers, medical practitioners and others, to cope with this increase [7]. The use of technology in the environment and on the person can therefore monitor and improve the patient’s quality of life [8].

Textile technologies, also known as “electronic textiles” and “e-textiles” [6,9,10,11] are fabrics made of filaments woven together in different ways that are capable of interacting with an external environment, such as the human body. “Smart textiles” is a further term [12,13,14], which is the one adopted by this research, highlighting the dedicated, or assistive, functions that regular textiles cannot fulfil [6].

Non-technological textiles have been used for many years for dementia care to stimulate activity and engage the person with activity [15]. With the increased availability of cheap sensors [16], wearable technology has taken off [17]. There is a stream of applications related to physiological sensors, but none specifically for a person with dementia for quality of life and cognitive assessment. Since the development of smaller computers, sensors that are small enough and/or unobtrusive enough to be worn have been a topic of interest in both the medical and electronics fields. Studies such as [18] have investigated various ways of implementing wearable (or otherwise portable) sensors, such as weaving sensor material into fabrics or sewn between two layers of cloth—which would then be used to make clothing [19].

This concept paper describes the technological and design considerations of a smart textile for use by the elderly and people in care homes. It also illustrates the key issues in the development of this smart textile. Important use cases for the smart textile are the improvement in the quality of life of the (care home) resident and developing methods for the appraisal of cognitive abilities. To do this, we review existing approaches and employ a novel architecture that demonstrates the ability to optimize the balance between personalization, functionality and complexity of development (and by extension, resultant cost). Textile implementations are constrained by the challenges of scaling the manufacture of personalized solutions [20]. While we do not implement specific new textile technology, we have created a system of working components sufficient to demonstrate the viability of this approach, a lab-based prototype, and the novelty of our approach is in the loosely coupled architecture, which enables the flexibility necessary for our complex use cases as a scalable solution.

### 1.2. Personalized Textiles for Quality of Life

Sensory and memory stimulation therapies have the potential to help improve many dementia-specific issues for individuals living in long-term care settings [21,22]. Techniques are very varied and include multisensory stimulation room sessions, aromatherapy and massage, dance/movement therapy [23], music therapy [24,25,26], and, related to this research, “comforting fabric artefacts”. These artefacts include aprons worn around the neck, fixed around the back, and quilts and smaller textiles designed to lie on a person’s lap while sitting. To stimulate the senses, they incorporate various features to play with, or “fiddle with” (hence the name “fiddle blankets”) such as zippers or fabric pieces. Recent additions include embedded microelectronics (sound, lights, and other feedback), which further add to the sensory experience [11,15,27]. Personalization can further add to the enjoyment of the textile, for instance, by creating textile representations of things, people, or memories that have meaning to the owner.

### 1.3. Gamification of Cognitive Tests in Textiles

Currently there are no definitive tests for assessing MCI [28,29] instead there is often applied an extensive neuropsychological battery of conventional diagnostic methods [29,30] with individual tests shown to have high susceptibility to false positive diagnostic errors [31,32]. The available methods require a clinician or psychologist to administer them to collect data, which introduces bias and increases cost, with most studies reported using a clinical consensus method [33], i.e., several clinicians.

Research into wearable artefacts for the sole purpose of monitoring health conditions [29,30] is extensive, and deals with topics such as the necessity of technology being either flexible or small enough to be unobtrusive [18,29]. “Serious Games” [31], games that are used for a utilitarian purpose, are an alternative way to explore the collection of data without a physician or psychologist present [32]. Games as treatment for various medical issues have been made [19], using wearable sensors both as an input device as well as for the purpose of monitoring those who play the game. The smart textiles can potentially measure cognitive ability without outside involvement, to reduce bias, and gather regular data over a longer time. Assessments of MCI within care facilities for the aged often occur annually [28], and so it is the intention that this device could collect data throughout the year to be analyzed by a psychologist on an ongoing basis. An example is the Wisconsin Card Sorting Test (WSCT, [34]), which could easily be implemented underneath the textile layer using the LED matrix and all the technological components. The WCST is commonly used by psychologists for memory assessment. Simple memory recall games or games such as “mastermind” [35] serve similar purposes, and again could be implemented simply using LEDs and switches. It is hoped that with further research, the data gathered could be used to create a baseline and, therefore, track cognitive ability over time, and, ultimately, be used in assessing cognitive decline. All the while entertaining the elderly patients in the care facility using a personalized, interactive, and entertaining textile.

## 2. Background Study

Recent developments in the field of microelectronics have allowed researchers to develop miniature circuits entailing sensing capability, front-end amplification, microcontroller functions, and radio transmission. Advances in material science have enabled the development of e-textile-based systems. With electronic thread, LEDs, and small microcontrollers, the garment itself becomes the computer. There are now systems that integrate sensing capability into garments, able to gather physiological and movement data. Similarly, there is a growing body of work focused on the application of wearable technology to monitor older adults and subjects with chronic conditions in care homes and community settings. This section reviews the relevant literature, covering assistive technology, wearable sensors (biomedical), and smart textiles. The latter section expands on the introduction to the use of smart textiles for quality of life and cognitive assessment, and the gamification of cognitive tests.

### 2.1. Assistive Technology

Intelligent assistive technology applications have been applied to dementia care and the aging population [7], for instance: the authors of [36] present a review of memory aid devices; the author of [8] reviews the use of AI to assist elders with cognitive impairment; the authors of [37] present information about game design for mental health care; the authors of [38] review the broader subject of ambient assisted living.

### 2.2. Wearable Technology

There are now nearly three decades of research since DARPA launched the Smart Modules Program to provide body worn computer systems to US soldiers [39]. Steady advances in sensor technology, microelectronics, telecommunication, and data analysis techniques have enabled the development and deployment of wearable systems for patients’ remote monitoring. Most of the applications of wearable sensors and systems focus on rehabilitation, health and wellness, safety, home rehabilitation, assessment of treatment efficacy, and early detection of disorders [19], and several are biomedical in nature, including personalized health [29] diagnostics, as well as monitoring applications. Physiological measures of interest in rehabilitation include physiological and biochemical sensing such as heart rate, respiratory rate, blood pressure, blood oxygen saturation, as well as motion sensing muscle activity, for instance [40]. Falls among the elderly frequently cause fatal and non-fatal injuries associated with a large amount of medical costs. Systems such as the fall detection system developed by [41] provide chronically ill and elderly people with the ability to extend an independent life. The system uses wearable wireless sensor network (WSN) technology developed for continuous monitoring of patients. When applied to a person, the term is body sensor network. Body sensor network systems can help people by providing healthcare services such as medical monitoring, memory enhancement, control of home appliances, medical data access, and communication in emergency situations [42]. Such continuous monitoring with wearable and also *implantable* body sensor networks can increase the early detection of emergency conditions and diseases in at risk patients and also provide a wide range of healthcare services for people with various degrees of cognitive and physical disabilities [42].

Wearable biomedical systems include: electrodermal activity (EDA) for distinguishing stress from cognitive load in an office environment [43]; magnetoencephalography (MEG) for non-invasive assessment of language function [44]; implanted wireless sensor networks [42]; data mining approaches using smartphones [45].

There are important design considerations however, which include unobtrusiveness, scalability, energy efficiency, and security [42]. Textile wearable computing products are not yet mainstream products; wearable products on the market are mainly electronic devices (such as watches and glasses) that can be attached to the body, so efforts in electronic textiles are still required.

Wearable Cognitive Assistance (WCA, [46]) represents a novel category of highly interactive and context-sensitive augmented reality applications that aim to amplify human cognition in both day-to-day tasks and professional settings. WCA systems were originally inspired by assistive use cases for people with some form of cognitive decline, either through aging or because of traumatic brain injuries. Cognitive decline can manifest itself in many ways, including the inability to recognize people, locations, and objects, loss of short- and long-term memory, and changes in behavior and appearance. Assistive systems include [47], and those with Google Glass devices to guide the user through a complex (cognitive) task, capturing the view of the wearer and combining with remote processing to perform real-time scene interpretation [48]. WCAs are anticipated to become a widely-used application class, in conjunction with emerging network infrastructures such as 5G that incorporate edge computing capabilities [49]. The key enabling technologies are sensor technology, communication technology, and data analysis techniques [19].

### 2.3. Smart Textiles

Soft, flexible fabrics with conductive properties can be used in place of conventional hard switches, keypads, buttons, or knobs. Made of conductive fibers, the fabrics are touch-sensitive and can also be used for proportional control of devices or pressure sensing—when pressure is applied, the resistance decreases until the fabric achieves metal-like conductivity. Conductive thread can be used to embroider electrical circuits. Smart textiles, electronic textiles, or e-textiles, are used to denote the class of fabric structures that integrate electronic elements with textiles [6,13]. Other commonly used terms include “intelligent fabrics”, “intelligent clothing”, “smart fabrics”, “wearable electronics”, and “textronics”. An interesting additional definition is that of “interactive textiles” or “i-textiles”[50], which is closer to the authors’ employment of the term smart textile. The term interactive textile places emphasis on the interactivity as the primary reason for the existence of the artefact, not simply integrating electronic elements into textile structures. E-textiles are divided by [51] into three subgroups: passive smart textiles—only able to sense the environment/user, based on sensors; active smart textiles—reactive sensing to stimuli from the environment, integrating an actuator function and a sensing device; very smart textiles—able to sense, react, and adapt their behavior to the given circumstances. This is a useful classification to explore, for instance, the degree of interactivity.

The smart textile field is rapidly developing, sitting at the intersection of disciplines such as engineering, computer science, health, and fashion. Smart textiles offer great potential to transform the practice of medicalization and health care [14], including sensory design for dementia care [15], and are becoming more mainstream [9,27,52]. The properties of e-textiles could include the use of various visual, tactile, sensory, and responsive elements to engage users in both physical and digital modes of interaction, thereby, potentially offering different and more intuitive ways of connecting with others, possibly strengthening existing relationships or accelerating the making of new ones. The authors of [53] propose e-textiles to enhance connectedness between lonely older adults. The move to smart textile medical devices also opens up the possibility of the creation of big data about patients’ bodies and lives [14,15].

#### 2.3.1. Sensors and Electronics Integrated into the Textile—“Sensorized Garments”

An important subset of e-textiles is those in which the monitoring devices are not only attached to the garment but sensors and electronics are also integrated into the fabric itself [9,13,54,55], so-called sensorized garments. Because a person wears the garment, for instance, for physiological monitoring, important considerations are the intrinsic rigidity of conductive materials, addressed with flexible electronics, and the mechanical deformations from stretching [13]. Such a continuous monitoring would be useful for several fields such as healthcare, fitness, and work.

One variant of this approach is to use fibers such as cotton, polyester, and lycra and coat them with metals such as silver, stainless steel, and copper. These fibers can be used to knit, weave, or embroider a garment, with the resultant electrical conductive properties [19]. Another variant is to either coat or print a conductive layer on a non-metallic substrate (fiber, yarn, or fabric) to make it conductive [19].

References [54,56] describe having the actual sensing structures produced in the textiles using processes that are compatible with the textile industry. The basic sensing elements (such as electrodes) need to be an integral part of the textile, utilizing a large area and the properties of the clothing. The emphasis is on sensing (not merely communication) structures in the textile, so that, with additional technology, these structures can implement various sensing principles, including capacitive, inductive, as well as biopotential principles [57].

This research envisions a clear separation between the various people creating the fabric, garments, electronics platforms, apps, and sensing fabric. The project targets incorporating generic, unstructured sensing layers into a fabric as it’s being produced. The simplest example of such a layer is a conductive coating applied to the entire surface. Another example is 2D grids of conductive threads woven into the fabric. More complex designs, such as two grids of conductive lines (woven or printed), separated by an insulating layer (that is, a potential array of resistive pressure sensors) are also possible. However, in all cases, the project assumes that the layers have no structure constraining them to specific, narrow apps or garments.

#### 2.3.2. Plugging Components into a Conductive Grid—The “Wearable Motherboard”

The previous research incorporated the electronic and sensing technologies during the actual manufacture of the textile. The technique presented in this section predates these latest developments significantly and hails from the late 1990s. It is described because it is a considerably different method and is conceptually similar to the architecture that we are proposing. The solution developed by [58] is similarly pervasive [59], but they achieve this by another route. They have developed a so-called Wearable Motherboard™ (WM) or Smart Shirt [60,61], a “platform” for sensors and monitoring devices that can unobtrusively monitor the health and wellbeing of individuals (directly and/or remotely). This can be considered the first responsive textile structure designed for a medical application, specifically for injury to soldiers, detecting bullet wounds, and special sensors and interconnects to monitor the body’s vital signs during combat conditions [56,62].

**Figure 1:**
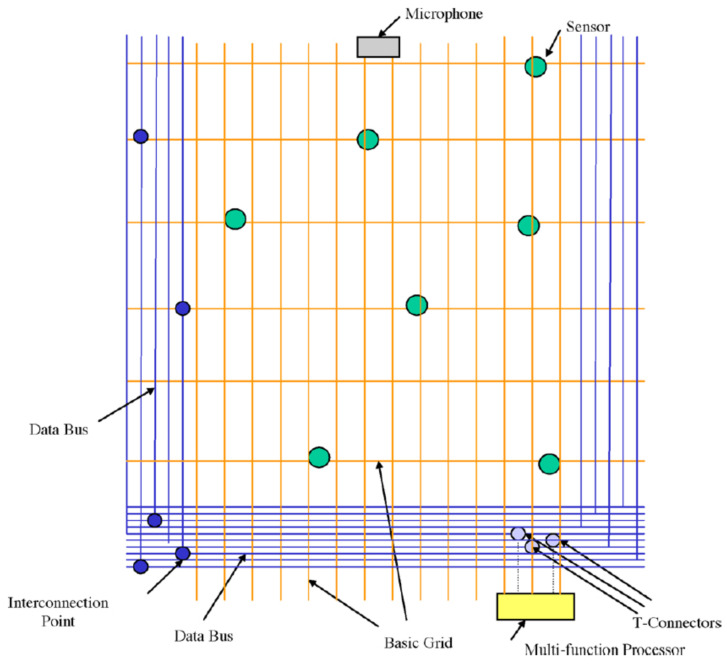
Architecture of the Smart Shirt/Wearable Motherboard, reprinted with permission from [57].

Figure 1 shows the architecture of the WM with a comfort or base fabric made from typical textile fibers (e.g., cotton, polyester, blends) providing the necessary physical infrastructure for the WM. Additionally, there are fiber optic and specialty fibers that serve as sensors and data buses to carry sensory information from the wearer to the monitoring devices; sensors for monitoring the respiration rate have been integrated into the structure. Sensors can be plugged in, for instance, a microphone to record the voice, and for monitoring a variety of vital signs including heart rate, respiration rate, electrocardiogram (EKG), body temperature, and pulse oximetry (SpO2).

The attraction of this approach is the customizability. Sensors can be attached and detached from certain designated “islands” on the garment [58].

### 2.4. Smart Textiles for Quality of Life

Sensory stimulation is an important tool for helping to improve quality of life and engagement for the aging population, especially people living with dementia [9,11,15,27]. Anecdotal evidence suggests that there is value in “comforting fabric artefacts”, enhanced by electronics (sound, lights, and other feedback), which engage and entertain these patients. New concepts are required to meet this challenge while keeping cost and recourses to a minimum [27].

These textiles can be covered with a selection of features, such as music players with fabric switches, games with LED’s, flexible, glass-free electrophoretic displays [63] for reminder systems, even fur that vibrates, and many other forms of engagement.

Music has long been an established treatment (intervention) for people with dementia [25]. This study found six key reasons for the continuation of music as a therapy, concluding that music goes further than reducing behavioral and psychological problems and helps maintain quality of life. Further studies to analyze the effect of music therapy on cognitive function in dementia are reported in [24]. This study sought to quantify music therapies in terms of six key areas. While their findings were inconclusive, their conclusion was that music therapy should continue, the problem being a lack of significant research into the area [24].

### 2.5. Smart Textiles for Cognitive Assessment

Recognition of cognitive impairment is an optimal stage to begin exploration of preventative therapies [1,6]. Testing for MCI is an arduous process that requires a physician or psychologist to administer the test to collect data, which introduces bias and increases cost. Previous tools for analyzing cognitive impairment include the Memory Impairment Screen [64,65], the General Practitioner Assessment of Cognition [54], The Montreal Cognitive Assessment (MoCA) [2], Mini-mental state [55], Telephone Interview of Cognitive impairment (TICSm, [66]), and the CogState Brief Battery [56]. Prior to the development of these tools the standard way to evaluate if a person had a cognitive impairment was through observation by clinical staff, interviews with specialists, and observation by family or caregivers [67]. Other tests exist, including memory-based tasks, such as the clock drawing test, where the individual is asked to draw a clock face with a given time [68], and the Wisconsin Card Sorting Test [69]. The use of technology has become more prevalent in the process of administering diagnosis tests to patients, e.g., CogState Brief Battery questions [56].

#### Gamification of Cognitive Tests

Games, as treatment for various medical issues, have been made [19,40,70] using wearable sensors both as an input device, as well as for the purpose of monitoring those who play the game. Games have been proposed as a viable therapy with research [62] showing that monitoring the rate of cognitive decline and its progress was an effective analytic tool, concluding that memory games were a useful tool in screening the early stages of cognitive decline. The gamification of cognitive tasks increases engagement and decreases the drop-out rate, increases willingness to continue each hour, accuracy, and can improve cognitive function, increase motivation, and willingness to participate [71]. However, it must be ensured that the scientific value of the test is not compromised through its gamification [64]. Reference [64] found that gamified cognitive tasks were generally equivalent to their non-gamified counterparts. Gamifying testing approaches presents itself as an ideal way to be able to gather the information in a simplified way that is enjoyable to the user, and that does not require the presence and cost of a skilled practitioner for administration. However, it must be acknowledged that a cost will still remain for the interpretation of the results.

### 2.6. Desirable Properties and Performance Requirements

Wearable technology must be both usable and affordable. The fabric in use must be light-weight and flexible. This means that ultrathin flexible circuit layouts or stretchable and flexible “circuit board fabrics” are needed (screen, power source, circuits, sensors). The power supply must be portable; therefore, it must be battery operated. Battery size/power consumption, charging methods, and charge time are valid areas of concern.

Features for e-textiles used for monitoring human parameters for extended periods of time, or use as intelligent personal assistants [51], include the following (taken mainly from [62,67]): wearability (comfort, including weight), connectability, durability, shape conformability, ease of care, ease of mending/maintainability, manufacturability, and affordability. Properties of the technological components include resistance to electromagnetic interference, thermal protection, electrical/optical conductivity, resistivity (of insulating layer), bending rigidity (replacing PCBs with flexible electronics), ease of diagnosing problems, ease of integration with sensors, processors (computing, wireless communication), monitors, and other equipment.

As noted in [65], in order for e-textiles to be affordable (cost of materials, manufacturing), they will need to be mass-produced, which is a difficult hurdle for new technology such as smart clothing and textile electronics. Current technology supports only special-purpose, low-volume textiles, garments, and electronics.

A requirement for an e-textile to be used in care home is that it needs to be launderable. The textile must be easy to fabricate, which implies a certain compatibility with standard manufacturing machinery. The electrical components require power, and so the ease of connection to a power source (battery charging and replacement) is an important consideration.

An important final consideration is that of data transmission, for instance through Bluetooth or Wi-Fi. There are important issues around privacy and security, and how to protect an individual’s personal information in data transmission and usage.

## 3. Design and Methodology

The aim of this project is to produce a hardware system to both entertain and monitor/assess the behavior of patients with dementia or its precursor of Mild Cognitive Impairment (MCI). The literature shows there are no similar systems. To this end a prototype system has been developed and is explained here. The smart textile (apron) includes a physical computing layer for music and games. Using microelectronics, sensors, and fabrics, the prototype aims to create provision for basic interactions with the sensory textile by incorporating light and sound effects as feedback when patients interact with different regions of the textile. The player (e.g., MP3 player [72]) and battery is stitched to a piece of fabric and connected to speakers and switches. A data logging system is included to record the patient’s behavioral patterns. This pattern data would include the location, frequency, and time of patient’s activities within the different textile areas.

Through a knowledge elicitation process the design of the textiles can be modified to incorporate detail pertaining to the client’s life story from data collected from family members. These elements are referred to as *persona metaphors*, artistic constructs that have psychological meaning for the person and which embody chosen aspects of their life experiences. Depending on the personalization of the apron, the switches will be “underneath” a particular persona metaphor, a scenario, for instance, a landscape, images of animals, or pictures of loved ones, playing when pressed, respectively, wind and water, or animal noises, or music and recordings from family members.

### 3.1. Design Considerations

Normal tactile switches are not suitable due to an operational requirement where the body of the switch must be held rigid so that the toggle may be operated. Fabrics, by nature, have no rigid structural integrity and, therefore, reliable operation has proven to be problematic. Secondly, to apply IP67 standards makes the tactile switch harder to operate due to the increased packaging and moisture protection, they are physically larger and take more force to operate.

**Figure 2:**
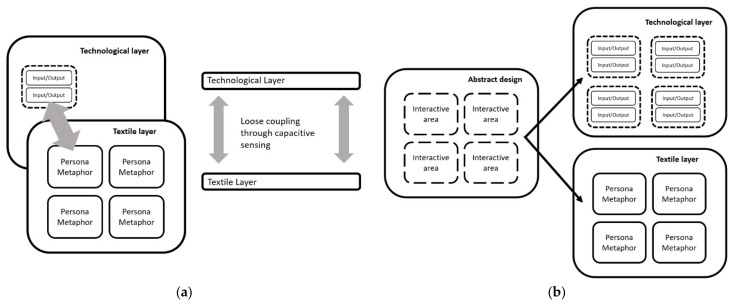
Overview of textile design: (**a**) Loose coupled layers—layers are aligned correctly through press studs and Velcro fastenings; (**b**) Implementing layers from design—a single design is sufficient to produce the layer with Input/Outputs. Input = capacitive sensors; Output = LEDs, vibrator motor, music player.

A key decision was to have a design with separate layers, shown in Figure 2.

The research implements a wide range of functionality by loosely coupling and concentrating (minimizing opposites) metaphors on the top layer, and engineering on the bottom layer (Figure 2a). Components in a loosely coupled system can be replaced with alternative implementations that provide the same services, and so this gives the solution the best flexibility.

### 3.2. Knowledge Elicitation

By using a loosely coupled design, the template (Figure 2b) can be used to create a beautiful upper layer of persona metaphors by textile experts, and the base layer is constructed by engineering processes. Part of the aim is to elicit life stories and embed those into these artefacts to enhance dignity and quality of life [73]. Quality of life is a broader concept than momentary enjoyment, and includes self-esteem, family issues, psychological distress, social support, relationships, and day-to-day concerns about the impacts of illness. The knowledge elicitation process, therefore, is similar to a life review, life reflections for palliative care patients [74]. Dignity therapy (DT) is an end-of-life psychological intervention that focuses on the creation of a life review document or legacy document to alleviate end-of-life distress [66] and that *is shared with others* [66]. This document is elicited by a trained therapist who invites people with life-limiting conditions to reflect on their life, find meaning, and leave messages for loved ones in a written, narrative document. In DT, a therapist invites a person facing death the opportunity to discuss aspects of their life they want remembered and share important messages during therapeutic conversations that are turned into a written legacy document [75,76]. DT uses 10 suggested questions to help guide the interview, including “Tell me a little about your life history, particularly the parts that you either remember most or think are the most important? What have you learned about life that you want to pass along to others?” [66].

### 3.3. Design

**Figure 3:**
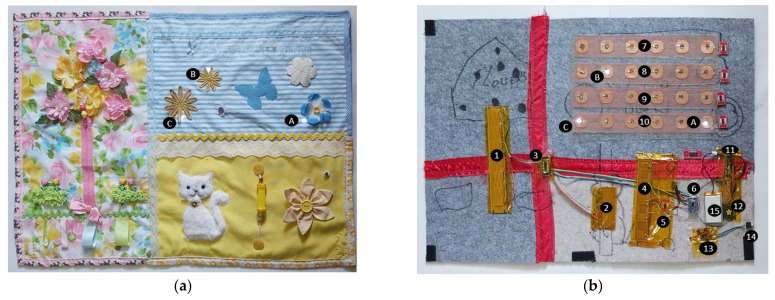
The two layers of the smart textile: (**a**) textile layer with persona metaphors—including cat, tree, sky; (**b**) technological layer where all the components are attached (refer to Table 1 for the list of components).

Figure 3a shows the developed smart textile made as a lap blanket. Its dimensions are 700 mm wide by 520 mm deep. The elements depict objects that are persona metaphors for a person; for example, this person liked their cat, and had birds and flowers and trees in their garden, which bought back pleasant memories, effecting a calming influence.

This project is to provide a universal technology layer (Figure 3b) underneath the top fabric (Figure 3a). The top fabric would be custom designed within the given layout of the technology layer to cater for individual user requirements (Figure 3a). This increases flexibility of the overall product, mitigating the costs associated with each artifact as a bespoke technical article.

The underlying technological layer (Figure 3b) is designed to house all electronics, sensors, LEDs, data-loggers, etc. The layer is made of thin felt sheeting, providing a soft, pliable barrier between the patient and the electronics, which reduces the noticeability of the embedded electronics components. The technology layer (Figure 3b) will be treated with a waterproof polymer coating before it is combined with the textile layer (Figure 3a) and sent out for user testing. A list of component types that are physically attached to this layer is listed in Table 1.

**Figure 4:**
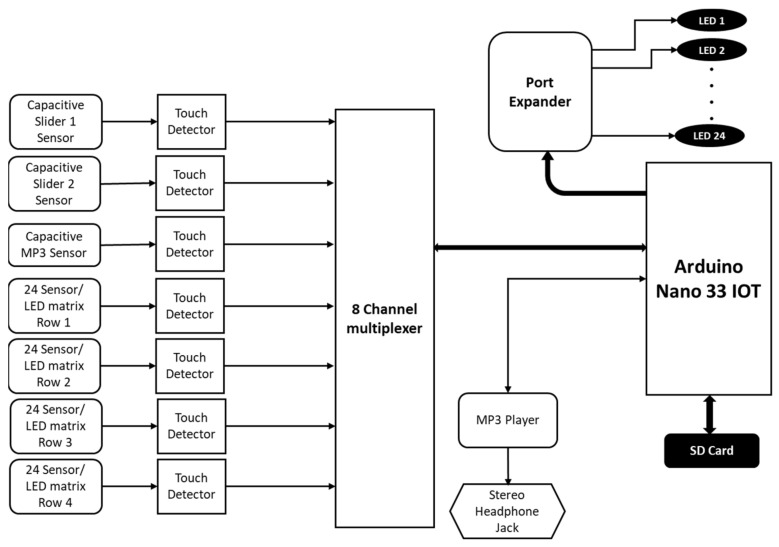
Schematic block diagram of the underlying technological layer in Figure 3b. All input/output devices are mapped to a specific location on the artifact. The selection of required switches/LEDs can be altered through the control program. This will allow an artisan the license to alter and shape the top layer to suit each patient’s personality as shown in Figure 3a.

**Figure A1:**
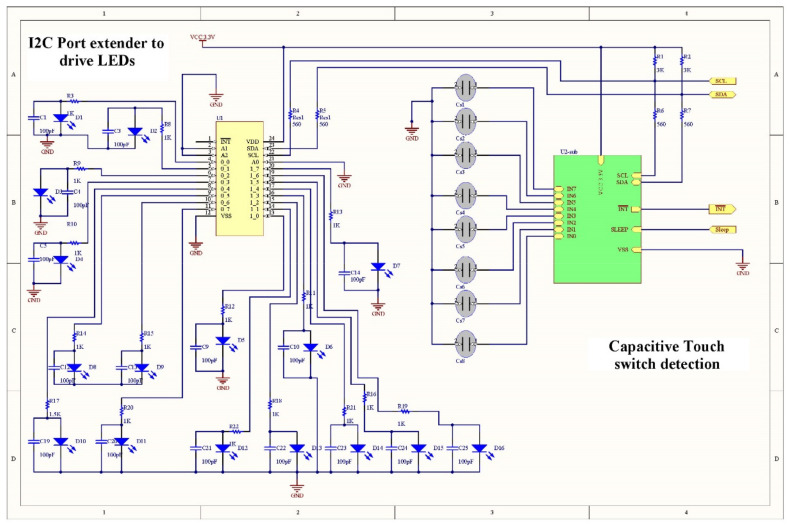
Digital switch and LED circuit diagram for textile interface.

The schematic block diagram of different components and their interconnections within the underlying technological layer (from Figure 3b), is presented in Figure 4. The capacitive sliding sensors and the capacitive touch sensors from each row of the LED array are connected to the 8-channel multiplexer (TCA9548A) through the touch detector (AT42QT1070 for the touch sensors and CAP1296 for the capacitive sliding sensors). The multiplexer communicates with the Arduino Nano 33 IOT. The Arduino [77] is also connected to (i) a 24 LED array through the port extender (NXP PCA9535), (ii) the MP3 player is connected to a stereo headphone jack, and (iii) an SD card for local data logging and storage. The detailed circuit layout of the capacitive touch sensors and the LED array connections are presented in Appendix A as Figure A1. All input/output devices (LEDs, capacitive touch, and sliding sensors, MP3 player, and the stereo jack) are mapped to a specific location on the artifact (Figure 3a). The selection of required switches/LEDs can be altered through appropriately programming the Arduino. This will allow an artisan the license to alter and shape the top layer to suit each patient’s needs.

The cat in Figure 3a attempts to mimic actual cat behavior using a capacitive slider sensor beneath the cat (item 2 in Figure 3b). The sensor generates an 8-bit binary number as an output that reflects the position of the hand during stroking along the length of the sensor. If the cat is touched, it may “meow”. When the cat is stroked at an acceptable rate, then it purrs, if the cat is stroked too fast, then it will show some annoyance by hissing, giving a more life-like experience to the textile. It is intended that the cat “purring” would be provided by the speaker, accessed through item 14 in Figure 3b. A final goal is to keep the textile as comfortable as possible. This includes size, weight, and the bulk from the electronics. This is important since the patients will have this on themselves when in use and cannot have anything that will cause them discomfort.

Music has long been held to be a useful tool in maintaining a persons’ cognitive ability and sense of themselves. The artifact is equipped with an integral MP3 player (item 13 in Figure 3b) to be loaded with the patients’ music of choice, and/or audio messages from family and friends. This system in the broader context can also be used for the relay of messages and prompts from the clinicians to the patient. The MP3 player is to be activated and controlled via the capacitive touch sensors shown in item 4 of Figure 3b.

As the system is microprocessor based, it lends itself to providing games for both entertainment (LED Game) and the monitoring of cognitive ability (Wisconsin Card Sorting Test/game, word games, memory games). The LED game utilizes LED sensor pairs (items 7 to 10 in Figure 3b), whereby the Arduino algorithm lights an LED, the user activates the associated switch to turn it off. The algorithm may light multiple LEDs at will. The game will collect response time data for each move. The speed and complexity of the required responses will change proportionally over time, thereby monitoring progress. Three such LEDs are lighted up in this case, A, B, and C in Figure 3b. The effect of these lighted up LEDs can be seen through the top layer—Figure 3a (A, Bm and C).

### 3.4. Digital Inputs (Switches)

Conventional tactile switches are not viable due to an operational requirement where one part (the body of the switch) must be held rigid so that the toggle may be operated. Fabrics by nature have no rigid structural integrity and, therefore, reliable operation proves to be problematic. Secondly to apply IP67 standards makes the tactile switch harder to operate due to the increased packaging and moisture protection, therefore, they are physically larger and take more force to operate.

The original fabric approach to switch manufacture was also discarded due to evidence of burn marks being found on the fabrics concerned. Numerous alternative switch designs were explored, collated from various community forums, open systems web sites, IC manufacturers application notes, and others in the public arena. Most of the designs utilize a single element, which means that the sensing element must be physically touched by a person. This in turn alters the capacitive balance that the sensor records as a touch. In a medical environment that introduces the possibility of electric shock and other undesirable interferences. Each switch must therefore conform to national standards (in this instance, Australian and ISO Standards) and be totally capacitive and isolated from human contact. Therefore, many of the designs including the original MPR121 were eliminated upon that basis.

Proximity detection is a viable alternative considered in this study. The proximity detectors include two types of capacitive touch sensors: (i) mutual capacitance and (ii) self-capacitive.

**Figure 5:**
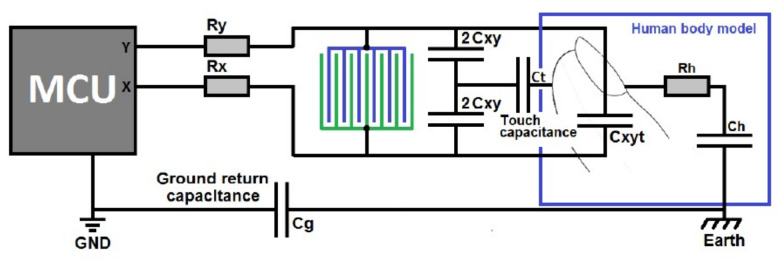
Circuit equivalence of a mutual capacitive touch sensor with an approaching finger [78].

In a mutual capacitive touch sensor, the capacitive effect is from the opposing side faces and the approaching finger. Figure 5 shows a pictorial view of the working principle of a mutual capacitive touch sensor, including the human body model (HBM). The system (Figure 5) uses two interleaved electrodes. When a touch is detected, the capacitance is modified. This touch contact is a complex arrangement where the HBM acts to increase the coupling between *X* and *Y* electrodes modelled by *C_XYT_*. Secondly, *C_T_* forms a ground return through *C_H_*, the HBM capacitance, and *C_G_* (ground return capacitance) causing an apparent decrease in capacitance. The equivalent XY capacitance for this circuit is given by (1). The larger the electrode spacing, the less sensitive the sensor will be. Optimal spacing is 2 mm greater than the fabric thickness at that specific location. The detection needs to take place at the exact location and should not be influenced by its neighbors.

(1) $$C_{eq}=\frac{4C_{XY}^{2}}{4C_{XY}+C_{f}}+C_{XYT}$$

where $C_{f}$ = *C_T_ C_H_* and *C_G_* in series.

**Figure 6:**
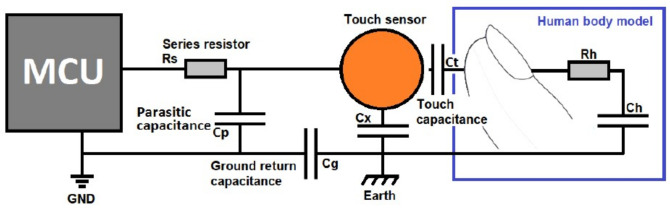
Circuit equivalence of a self-capacitance touch sensor with an approaching finger [78].

Mutual capacitive touch sensors are challenging to construct. As a result, this study builds and employs self-capacitance touch sensors. Figure 6 shows a pictorial view of the self-capacitance technology including the HBM.

The capacitance of the sensor is comprised of parasitic capacitance (*C_P_*) in parallel with ground return capacitances (*C_X_* and *C_G_* in series). When a finger approaches, the human body model is applied, which increases the capacitance of the sensor through the series capacitance *C_T_* and *C_H_* in parallel with *C_X_*. The value of *C_T_* (1 pF) is much smaller than both *C_H_* and *C_G_* (100 pF each). It is accepted that the HBM capacitance is between 100 and 200 pF [75]. The total capacitance can be calculated using (2).

(2) $$\frac{1}{C_{total}}=\frac{1}{C_{T}}+\frac{1}{C_{H}}+\frac{1}{C_{G}}$$

Three different types of self-capacitance touch sensors were constructed and used in the technological layer (Figure 3b). They are: (i) Capacitive Touch Sensor 1 (CTS1)—for switching the LEDs, as part of gamification (items 7,8,9,10 in Figure 3b), (ii) Capacitive Touch Sensor 2 (CTS2)—for controlling the MP3 music player based on user input (item 4 in Figure 3b), and (iii) Capacitive Touch Slider Sensor 1 (CTSS1)—for interactive playing with the cat and tree (item 1,2 in Figure 3b).

**Figure 7:**
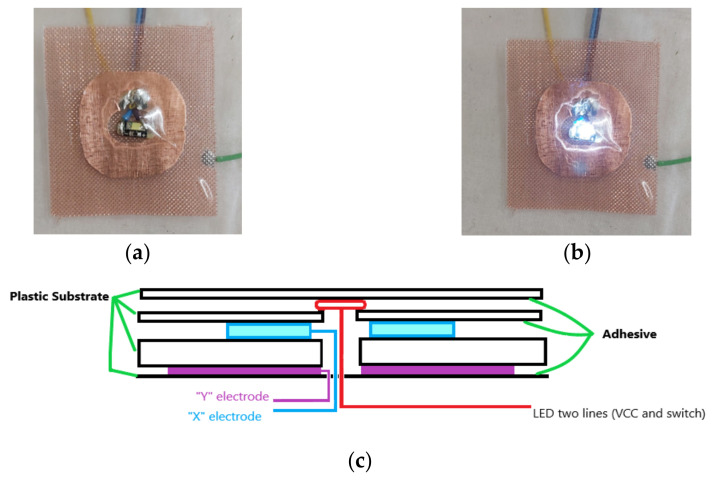
Capacitive touch sensor (Capacitive Touch Sensor 1—CTS1) with integrated LED (items 7,8,9,10 in Figure 3b) showing (**a**) LED not lighted, (**b**) LED lighted, and (**c**) construction principle.

Self-capacitance touch sensors run the risk of inadvertent activation from the rear resulting in a patients’ leg movement giving a false detection. To prevent rear detection, a guard electrode is used during the construction in the rear of CTS1, CTS2, and CTS3. The guard electrode is driven at the same potential as the touch sensor. The electrode is built as 50% copper mesh and sits behind the touch sensor to shield the sensor from responding to any approach from the rear. This can be viewed in both Figure 7a,b.

The picture of the constructed CTS1 is shown in Figure 7a with the LED switched off, and Figure 7b with the LED switched on. The 50% copper mesh guard electrodes can also be seen in both Figure 7a,b. The construction principle of CTS1 is presented in Figure 7c. These CTS1s can be identified in the underlying technology layer (Figure 3b) as items 7,8,9,10.

**Figure 8:**
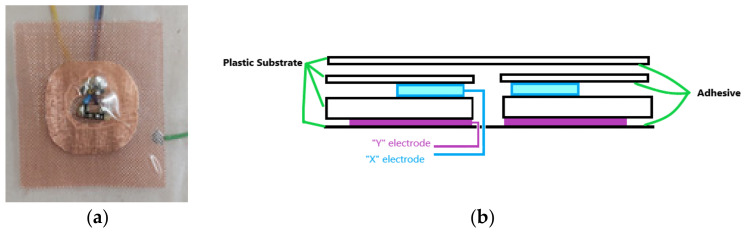
(**a**) Capacitive touch sensor (Capacitive Touch Sensor 2—CTS2) (item 4 in Figure 3b), and (**b**) construction principle.

The picture of CTS2, identified in the underlying technology layer (Figure 3b) as item 4, is shown in Figure 8a, including the guard electrode. The construction principle of CTS2 (Figure 8b) is the same as that of CTS1 (Figure 7c), without the integrated LED.

The CTS3 (identified as items 1 and 2 in the underlying technology layer in Figure 3b) is constructed as a series of identical capacitive touch sensor blocks and guard electrodes to prevent sensor responding to any touch from the rear. As the finger slides down from one block to another, a change in capacitance is detected, which is correlated to find the position of the touch. The dimension of CTS3 in item 1 (Figure 3b) is 180 mm in length and 20 mm wide. The CTS3 in item 2 (Figure 3b) is 75 mm in length and 20 mm wide.

### 3.5. Data Logging and Communication

The Nano 33 IoT facilitates recording of all the user interactions and is saved in (i) SD card (item 12 in Figure 3b) and (ii) file server in the local Wi-Fi network. In the eventuality of a network failure or any other reasons for unavailability, the integrity of the data is maintained as a result of being on the product storage using an SD card. Given the large body of literature concerning the acquisition, processing, and analysis of data from body wearable sensors, data security has received a lot of attention [70,79,80]. To create a secure infrastructure, the data are transferred using appropriate encryption protocols and authentication mechanism. The Nano 33 IoT contains an in-build crypto authentication co-processor (ATECC608A). It provides secure point to point data transfer over wireless.

The type of data collected depends on the nature of the interactions.

(a) From the MP3 player—data collected are (i) timestamp, (ii) track name, (iii) track length, and (iv) play time.
(b) From the games area—data collected are timestamp corresponding to (i) turning on and off of the LEDs (items 7,8,9,10 in Figure 3b) and (ii) detection in the touch sensors (items 7,8,9,10 in Figure 3b).
(c) Cat and the tree—data collected are timestamp corresponding to the detection on the capacitive sliding sensors (items 1 and 2 in Figure 3b), including frequency.

The timestamp is generated through the real-time clock (RTC) integrated with Nano 33 IoT.

### 3.6. Energy Consumption

For the initial prototype, a Raspberry Pi 4 was used as the processor for its ability to perform a wide range of tasks. The Raspberry Pi 4 is rated at 5 V and 3 A. However, the processor operates only at 3.3 V. Thus, the input voltage requirement is set at 3.3 V with maximum current of 4 A. This is broken down in the maximum current consumption profile presented below.

- LEDs, 20 × 20 mA = 0.4 A
- Neo-pixels RGB, 10 × 60 mA = 0.6 A
- Raspberry Pi 4, 3 A

Polymer lithium batteries, rated at 3.7 V and 4400 mAh, are utilized in the prototype. However, with the battery power cables only rated at 1 A discharge rate, the protype had to be powered from the desktop supply. To improve the portability of the prototype, the processor is being changed to Arduino Nano 33 IoT board, with power rating of 5 V and 0.2 A (item 11, Figure 3b). The LEDs have also been replaced with ADAFruit sequins, each having a current draw at 3.3 V of 5 mA.

With the Arduino, the maximum current drawn is now only 0.9 A. The consumption profile of the blanket is as follows.

- LEDs, 20 × 5 mA = 0.1 A
- Neo-pixels RGB, 10 × 60 mA = 0.6 A
- Arduino Nano 33 IoT board, 0.2 A

To provide a better understanding of the energy consumption and operational battery life of the blanket with the new Arduino Nano 33 IoT board, the following three scenarios are considered. Based on the scenarios, an estimated operational battery life of the polymer lithium battery (rated at 3.7 V and 4400 mAh) is also highlighted.

- Scenario 1—The prototype is used for gaming with LEDs illuminated, stroking the cat, and playing the MP3 player through the Bluetooth speaker. The current consumed would be 300 mA maximum. This would give an estimated operational battery life of 14 h.
- Scenario 2—Just playing the MP3 player through the Bluetooth speaker. The current consumed would be 200 mA. This would give an estimated operational battery life of 22 h.
- Scenario 3—The system is in standby mode. The current consumed would be 10 mA. This would give an estimated operational battery life of 440 h.

The current polymer lithium battery needs to be recharged off the product. However, a wireless charging option can be incorporated in the future, utilizing a commercially available wireless battery charger that can deliver up to 12 V at 5 A. The charging regime can then be built into patient chairs (retrofitted) to keep the power sources charged. However, this option is out of the scope of this current work.

### 3.7. Laundering in Care Home Setting

A further benefit of the loosely coupled layers is the necessity to launder the textile. Technology that embeds electronic functionality through either coating the textile with smart or functional material, or embedding miniature MEMS sensors in the yarns or fabric [11] will require this fabric to be washed (repeatedly), thus reducing its lifespan considerably. Technology that inserts electronic functionality into “islands” on the textile, while removing the core components still leaves the connectors, again deteriorating through washing. The loosely coupled solution proposed here has the benefit that the most likely or most soiled component is the top layer, which is solely textile, while the technological components in the technological layer (also containing the removeable battery) will be protected using a flexible molded polymer layering process. This will encapsulate the electronic components and also protect the circuitry and sensors from external elements.

## 4. Results and Discussion

### 4.1. Capacitive Slider Switch Sensor Performance

**Figure 9:**
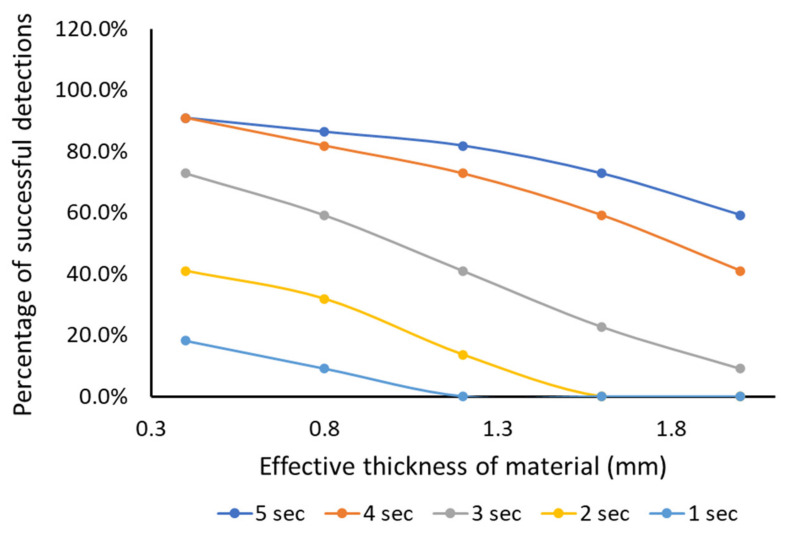
Performance of capacitive slider sensor in the smart textile (items 1 and 2 in Figure 3b).

The performance of the capacitive slider switch sensor against the effective thickness the of material above the sensor for different stroking time is presented in Figure 9. The performance of the sensor is measured in terms of the percentage of successful detection of a binary output number in the correct sequence, pre-set in the program.

For a stroking time of 5 s, as the effective material thickness increases from 0.4 mm, the percentage of successful detections of the correct sequence reduces by 33% from 90%. The reduction only indicates the sensor is failing to generate the pre-set correct sequence.

For a stroking time of 4 s, as the effective material thickness increases from 0.4 mm, the percentage of successful detections of correct sequence reduces by 55% from 90%.

For a stroking time of 3 s, as the effective material thickness increases from 0.4 mm, the percentage of successful detection of correct sequence reduces by 87% from 72%.

For a stroking time of 2 s, the percentage of successful detections of correct sequence reduces to 0% as the effective material thickness reaches 1.6 mm.

For a stroking time of 1 s, the percentage of successful detections of a correct sequence reduces to 0% as the effective material thickness reaches 1.2 mm.

It is observed that the faster the stroking time, the percentage of the successful detection of a correct sequence significantly reduces, and the performance degrades much faster as the effective material thickness over the sensor increases. The recommended effective material thickness is 2 mm thickness with 5 s stroking time.

It is to be noted here that the cat circuitry is programmed to only respond when the correct sequence (pre-set in the program) is detected from the sensor, not just when it is touched or stroked differently. This should emulate the behaviour of a real cat, which would only react when they receive the right affection. For this particular programmed setup and accurate detection of the correct sequence, the recommended stroking time is 5 s for the effective material thickness of 2 mm. The response frequency can be modified at the software level, depending on the user preference.

### 4.2. Detection Performance of Capacitive Touch Sensors

**Figure 10:**
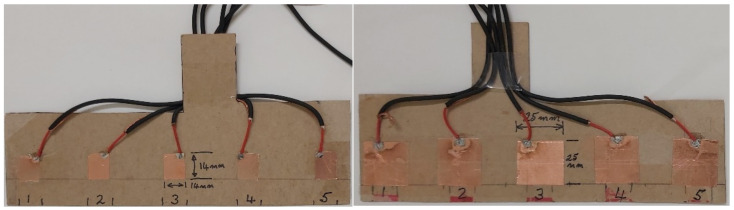
Capacitive touch sensor setup in blocks of both 14 mm × 14 mm and 25 mm × 25 mm detector plates.

Figure 10 shows switch blocks used in experimentation to determine sensitivity and detection range; they were constructed in blocks of both 14 mm × 14 mm and 25 mm × 25 mm detector plates (Figure 10). This was the basis of choosing the size of the capacitive touch sensors—CTS1 and CTS2. For testing purposes, cardboard was used as a guard electrode to prevent the sensors responding to any approach from behind.

**Figure 11:**
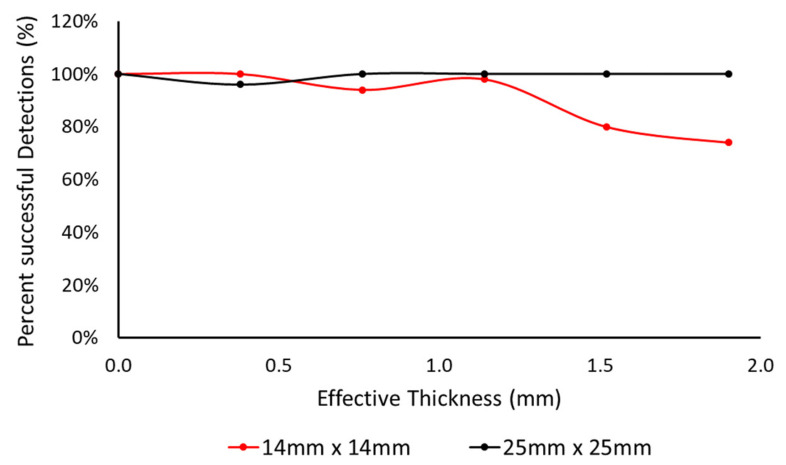
Performance of capacitive touch sensor setup in blocks of both 14 mm × 14 mm and 25 mm × 25 mm detector plates.

The detection performance of the sensor blocks was measured against effective thickness of the covering material. The Atmel AT42QT10 family detector chip was used for processing the sensor signals. The experiment was conducted by fixing the switch into a stable position and applying a different measured thickness of material placed on top of the sensor (0 to 1.9 mm). Fifty touches, at an average rate of one per second, were applied, and legitimate detections were measured through the Arduino ADC input channel. The detection performance with varying fabric thickness is presented in Figure 11. The bigger surface area of the 25 mm × 25 mm detector plates provided significantly better performance in comparison to the smaller 14 mm × 14 mm. With the different effective material thickness, the 25 mm × 25 mm detector places provided an average of 99% accuracy in detecting the passes. The number of correct detections dropped to 91% for the smaller sensor plates. The 25 mm × 25 mm detector plates were, thus, ultimately used for the construction of CTS1 and CTS2.

**Figure 12:**
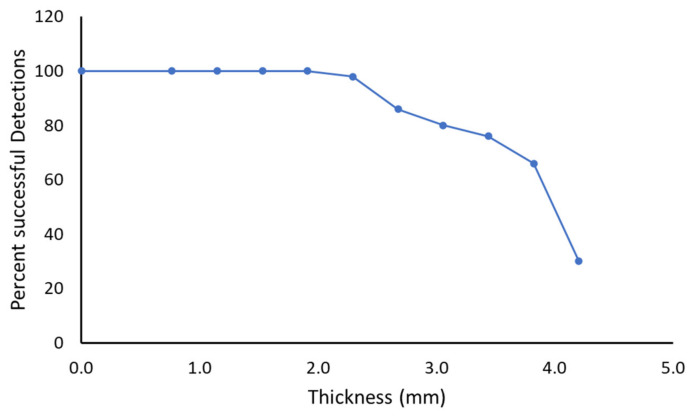
Performance of the constructed capacitive touch sensors in the smart textile (items 4,7,8,9,10 in Figure 3b).

The detection performance of the constructed capacitive touch sensors (CTS1 and CTS2) was also tested using a similar testing condition as described above. The effective thickness of the covering material was varied between 0 and 4.18 mm and the performance is plotted in Figure 12. The detection percentage was at 100% up to 2 mm effective material thickness. As the effective thickness increased, the percentage of correct detection reduced significantly. At 3.1 mm, 80% of the passes were correctly detected and at 4.2 mm only 30% of the passes were detected correctly.

## 5. Conclusions

Two very significant approaches to e-textiles are the sensing fabric (where the electronic and sensing technologies are incorporated during the actual manufacture of the textile) and the plug-and-play wearable motherboard. The former results in a robust fabric, mass producible with industrial processes, such as weaving and knitting, requiring bespoke crafting into high-level (or human level) applications. The latter offers a very flexible architecture with designated areas that can be embedded with technology. We present a third approach, through a prototype architecture that is comprised of loosely coupled layers, separating the textile from the technology layer, which offers significant benefits in flexibility. Our approach shares certain characteristics with the latter. By utilizing capacitive sensing, we can loosely couple the technology from the textile, enabling separation and, hence, the division of labor between the technologists and textile designers. If the technology and textile were embedded in one layer, both would need to be present to develop the artefact.

A point to note is that the architecture works well with simple interaction, and the solution is easily loosely coupled. Simple Input/Output technology such as switches and LEDs can easily be embedded under ever changing textile layers. The textile layer can be designed independently of the technology layer, as long as the abstract design is known. Therefore, personalization can be quickly and easily generated for new people. However, where there is more complex interaction (e.g., LCD display), the solution is more strongly coupled.

The use case presented is that of improving the lives of residents in care homes and assisted living through increasing quality of life, knowledge elicitation process and embodiment of life experiences, and cognitive appraisal. Future work involves user testing of the final smart textile and consideration of a broader set of use cases to determine the limits of this approach.

## Figures and Tables

**Table 1:** Component list of the underlying technological layer in Figure 3b.

Location Number in Figure 3b	Description	Location Number in Figure 3b	Description
1, 2	Capacitive sliding sensors	11	Arduino Nano 33 IoT
3	Dual detector for the capacitive sliding sensors	12	SD card for primary data logging
4	Capacitive touch sensors for switching the MP3 player	13	MP3 player
5	Detector for the capacitive touch sensors for switching the MP3 player	14	3.5 mm stereo jack for headphones
6	8-channel multiplexer	15	Battery pack
7, 8, 9, 10	LED array with capacitive touch sensors for switching the gaming. It also includes the detectors for the sensors		

## Appendix A

The digital switch and LED circuit diagram for the textile is shown in Figure A1.
